# Knowledge and Interdisciplinary Communication of Gastroenterologists and Dentists in the Netherlands About Gastrointestinal Diseases With Oral Manifestations

**DOI:** 10.1093/crocol/otac006

**Published:** 2022-02-28

**Authors:** Christopher X W Tan, Henk S Brand, Oumaima Qaddour, Paulette M L van der Bijl, Nanne K H De Boer, Tymour Forouzanfar, Jan G A M de Visscher

**Affiliations:** 1 Department of Oral and Maxillofacial Surgery/Oral Pathology, Amsterdam University Medical Centre/Academic Centre for Dentistry Amsterdam (ACTA), Amsterdam, The Netherlands; 2 Department of Oral Biochemistry, Academic Centre for Dentistry Amsterdam (ACTA), Amsterdam, The Netherlands; 3 Department of Gastroenterology and Hepatology, Amsterdam University Medical Centre, VU University, AGEM Research Institute, Amsterdam, The Netherlands

**Keywords:** inflammatory bowel diseases, Crohn’s disease, ulcerative colitis, oral manifestations of inflammatory bowel diseases, interdisciplinary communication/consultation

## Abstract

**Background:**

Gastrointestinal diseases can have oral manifestations. The aim of this study was to investigate the knowledge of gastroenterologists and dentists about gastrointestinal diseases with oral manifestations and to assess the frequency, extent and content of communication between gastroenterologists and oral healthcare professionals.

**Methods:**

Separate questionnaires were developed and sent to all 523 gastroenterologists and a random selection of 500 dentists in the Netherlands. Both questionnaires contained questions about demographic characteristics of the participants, 10 statements about gastrointestinal diseases with possible oral manifestations and questions about the communication between gastroenterologists and oral healthcare professionals. Additionally, the questionnaire for gastroenterologists contained 9 statements about general dentistry and the questionnaire for dentist had 9 questions about gastrointestinal diseases.

**Results:**

Gastroenterologists answered 47.6% ± 31.9% of the questions correct about gastrointestinal diseases with possible oral manifestations and 57.5% ± 27.9% of the questions correct about general dentistry. Dentists answered 26.6% ± 20.5% of the questions correct about possible oral manifestations of gastrointestinal diseases and 50.3% ± 18.7% of the questions correct about gastrointestinal diseases. Gastroenterologists and dentists valued interdisciplinary consultation as very useful with scores of 4.07 ± 0.70 and 4.67 ± 0.49 on a 5-point Likert scale, respectively, but the frequency of consultation was considered insufficiently with a mean score of 2.88 ± 1.01 and 2.24 ± 1.05 on a 5-point Likert scale, respectively.

**Conclusions:**

This study suggests that the knowledge of gastroenterologists and dentists about gastrointestinal diseases with oral manifestations could be improved. Interdisciplinary consultation was considered valuable for the optimal treatment of their patients but was assessed as insufficient.

## Introduction

Crohn’s disease, ulcerative colitis, Peutz–Jeghers syndrome, and celiac disease are gastrointestinal diseases that may be associated with oral manifestations. There is a wide variety of more or less specific oral conditions including aphthae, cobblestones in the buccal mucosa, diffuse swelling of the lips, buccal mucosa and face, pyostomatitis vegetans, hyperpigmentation on the lips and oral mucosa, enamel defects, and xerostomia. Some or a combination of these oral diseases can have a long-time negative impact on the quality of life in patients.^1–5^ Since gastrointestinal symptoms are often predominant, the first consultation is usually by a gastroenterologist where awareness of the possible presence of associated oral health problems is important. Oral manifestations may sometimes precede intestinal disease^6^ and approximately 30% of the patients continue to manifest oral lesions despite control of their intestinal disease activity.^7,8^

Previous studies have shown that dental professionals were knowledgeable about oral–systemic health associations, but had mixed feelings about translating this information into the dental practice.^9,10^ On the other hand, recent surveys among general practitioners concluded that their knowledge about the relation between periodontal diseases and systemic disorders needed to be improved.^11,12^ Medical consultations by dentists still seem to be rare. However, when such consultations take place they frequently result in an alteration of the dental treatment plan.^13^ This suggests that knowledge about oral manifestations of gastrointestinal diseases is important for both dentists and gastroenterologists, and good communication between these healthcare professionals is essential for optimal patient care.

Therefore, the aim of this study was to investigate the knowledge of gastroenterologists and dentists in the Netherlands about gastrointestinal diseases with oral manifestations. Secondary, we wanted to assess the frequency, extent and content of the communication between gastroenterologists and oral healthcare professionals in the Netherlands and what value is attached to this.

## Materials and Methods

### Questionnaires

Two separate questionnaires were developed, 1 for gastroenterologists and 1 for dentists. Both questionnaires consisted of 4 parts and participants were asked to answer the questions with their current knowledge without consulting scientific literature or internet. The first part was the same for both professions and contained general questions about demographics, work conditions, and the extent and content of patient care. The second part for gastroenterologists contained questions regarding the frequency, content, and their opinion on the importance and value of the communication between gastroenterologists and various oral healthcare professionals (dentists, oral hygienists, and oral and maxillofacial surgeons). The second part for dentists had questions about various aspects of the communication with gastroenterologists. The third part of the questionnaire for gastroenterologists explored their knowledge about dentistry in general and gastrointestinal diseases with oral manifestations, while the third part of the questionnaire for dentists explored their knowledge about gastrointestinal diseases in general and gastrointestinal diseases with oral manifestations. These statements could be answered with “correct,” “incorrect,” or “don’t know.” The final part of the questionnaire explored the opinion of the gastroenterologists and dentists regarding the knowledge of, respectively, oral health and gastrointestinal diseases and about the sources of information they use. The answers for these items were based on recent reviews of the relation between oral health and gastrointestinal diseases.^1–4^ Preliminary versions of the questionnaires were tested on 3 gastroenterologists and 4 dentists for understanding and clarity. Their feedback led to some minor adjustments of the questionnaires.

### Study Population

The final versions of the self-developed questionnaires were distributed by mail among all 523 gastroenterologists in the Netherlands. For the dentists, a random selection of 500 dentists was taken from the member guide of the dental association in the Netherlands (about 9000 members). When a randomly selected member was an oral and maxillofacial surgeon or an orthodontist, this member was not included but replaced by another general dentist. The envelopes contained the questionnaire, a letter with an explanation of the study and a prepaid envelope to return the questionnaire free of charge and anonymously. The questionnaires were mailed only once.

### Statistical Analysis

Data are expressed as mean ± SD or percentages and were analyzed statistically with IBM SPSS Statistics for Windows Version 25.0 (IBM) using Chi2 tests, Mann–Whitney *U*-tests, and ANOVA tests. For statistical analysis of the answers to statements, the gastroenterologists were dichotomized based on the median year of graduation, 2006, into 2 subgroups (≤2006 versus > 2006), and stratified according to gender and whether they consulted an oral healthcare professional or not. The dentists were stratified into subgroups according to gender and the way they obtained their current knowledge (their studies at dental school, textbooks, or scientific articles). Answers on a Likert scale of 1–5 were divided into insufficient (scores 1 and 2), neutral (score 3), and sufficient (scores 4 and 5). All significance levels were set at 0.05.

### Ethical Considerations

This study followed the Declaration of Helsinki on medical protocol and ethics and the data were collected in accordance to the guidelines of the Medical Ethical Committee of the VU University Centre. The Medical Ethical Review Committee of the VU University confirmed that the Medical Research Involving Human Subjects Act (WMO) does not apply to this study and therefore an institutional review board approval was not applicable.

## Results

### Gastroenterologists

A total of 107 gastroenterologists returned the questionnaire, resulting in a response rate of 20.5%. There were 32.7% females and 67.3% males and the average age of the respondents was 48.2 ± 9.7 years (range 32–65 years). The average year of graduation was 2005 ± 9 years ranging from 1984 to 2018. On average, the gastroenterologists worked 42.3 ± 9.3 hours per week (range 16–80 hours) and the mean number of patients consulted per week was 72 ± 32 (range 1–180). These data are summarized in Table 1.

**Table 1.** Demographic characteristics of gastroenterologists (*n* = 107) and dentists (*n* = 93).

	Gastroenterologists	Dentists
Male	72 (67.3%)	56 (60.2%)
Female	35 (32.7%)	36 (38.7%)
Gender not reported	0 (0.0%)	1 (1.1%)
Mean age (years)	48.2 ± 9.7	48.3 ± 12.8
Year of graduation	2005 ± 9	1993 ± 13
Working hours per week	42.3 ± 9.3	32.8 ± 7.7
Consulted patients per week	72 ± 32	88.5 ± 44

In the period of the last 5 years, 52.4% (*n* = 58) of the gastroenterologists contacted an oral health professional; 86.2% of these 58 gastroenterologists consulted an oral and maxillofacial surgeon, 37.9% a dentist and 5.2% an oral hygienist (multiple answers were possible). The mean number of contacts in the last 5 years (*n* = 56) was 11.9 ± 23.8 with a range of 1–125. 67.2% of the consultations were because of oral symptoms which could be related to gastrointestinal diseases and 34.5% of the consultations were initiated to discuss the possible influence of these oral symptoms on the treatment plan. 8.4% of the gastroenterologists always asked patients about oral manifestations during the first consultation whereas 5.6% never asked patients about oral manifestations. 6.5% of the gastroenterologist always examined the oral cavity during the first consultation while 80.4% of the gastroenterologist examined the oral cavity only when the patient reported complaints. When oral disease was present, the gastroenterologists valued consultation of an oral healthcare professional as very useful for the treatment of their patients with a mean score of 4.07 ± 0.70 on a 5-point Likert scale. Gender and year of graduation had no significant effect on these scores (Mann–Whitney *U*-test *P* = .130 and *P* = .633, respectively). Gastroenterologist noted that there was not enough structured contact between gastroenterologists and oral healthcare professionals with a mean score of 2.88 ± 1.01 on a 5-point Likert scale and they indicated that communication between the 2 disciplines is useful for optimal treatment of their patients (4.37 ± 0.71). 46.5% of the gastroenterologists suggested that the curricula of dental schools and medical schools should be more aligned to improve the communication between the 2 disciplines.

Table 2 shows the answers of the gastroenterologists on 9 statements about general dentistry. They answered 57.5% ± 27.9% of the questions correct and 23.9% ± 19.1% were answered with “don’t know.” The percentages of correct answered questions varied considerable for the different questions. Statements on function of dentures and lack of saliva were frequently answered correctly whereas statements on the number of teeth of children and the potential role of bacteria in dental erosion were answered poorly. Table 3 shows the answers of the gastroenterologists on 10 statements about gastrointestinal diseases with possible oral manifestations. They answered 47.6% ± 31.9% of the questions correct and 34.5% ± 19.3% of the questions were answered with “don’t know.” Gastroenterologists who had graduated after 2006 answered more statements correct about gastrointestinal diseases with possible oral manifestations than their colleagues who had graduated in 2006 or before, but this difference did not reach statistical significance (Mann–Whitney *U*-test, *P* = .796).

**Table 2.** Answers of gastroenterologists on statements about general dentistry.

	True	False	Don’t know
Dental caries is caused by acid producing bacteria.	**84 (78.5%)**	11 (10.3%)	12 (11.2%)
Dental erosion in caused by acid producing bacteria.	68 (63.6%)	**22 (20.6%)**	17 (15.9%)
Smoking has a positive effect on periodontitis.	9 (8.4%)	**89 (83.2%)**	9 (8.4%)
A complete adult dentition has 28 teeth (wisdom teeth excluded).	**54 (50.9%)**	19 (17.9%)	13 (31.1%)
A complete children’s dentition has 24 teeth.	36 (34.3%)	**20 (19.0%)**	49 (46.7%)
The function of a conventional denture is comparable with the natural dentition.	3 (2.8%)	**93 (86.9%)**	11 (10.3%)
Decreased salivation increases the chance of developing dental caries.	**92 (86%)**	2 (1.9%)	13 (12.1%)
Dental erosion is caused by an acid rich diet.	**63 (58.9%)**	26 (24.3%)	18 (16.8%)
Removal of a molar in the lower jaw can cause damage to the inferior alveolar nerve.	**36 (33.6%)**	4 (3.7%)	67 (62.6%)

The correct answer to the statement is indicated in bold (*n* = 105–107, not every question was answered by all gastroenterologist).

**Table 3.** Answers of gastroenterologists on statements about gastrointestinal diseases with possible oral manifestations.

	True	False	Don’t know
The prevalence of dental caries is higher in patients with Crohn’s disease than in patients with ulcerative colitis.	**7 (6.8%)**	37 (35.9%)	59 (57.3%)
Pyostomatitis vegetans is more prevalent in patients with ulcerative colitis.	**11 (10.7%)**	52 (50.5%)	40 (38.8%)
Cobblestoning in the oral cavity occur in patients with ulcerative colitis.	3 (2.9%)	**76 (73.1%)**	25 (24%)
Linear ulcerations in the oral cavity can occur in patients with Crohn’s disease.	**93 (89.4%)**	2 (1.9%)	9 (8.7%)
The prevalence of gingivitis/periodontitis is higher in patients with ulcerative colitis.	**21 (21%)**	23 (23%)	56 (56%)
Diffuse swelling of the lips and buccal mucosa occur in patients with Crohn’s disease.	**52 (50.5%)**	7 (6.8%)	44 (42.7%)
Halitosis and changes in taste is a common symptom in patients with celiac disease.	32 (31.1%)	**26 (25.2%)**	45 (43.7%)
Enamel defects are more common in patients with celiac disease.	**42 (40.4%)**	8 (7.7%)	54 (51.9%)
Aphthous ulcerations are more common in patients with celiac disease than in patients with Crohn’s disease.	20 (19.2%)	**68 (65.4%)**	16 (15.4%)
Oral characteristics of Peutz–Jeghers syndrome are distinct mucocutaneous pigmentations on the lips and oral mucosa.	**97 (93.3%)**	0 (0%)	7 (6.7%)

The correct answer to the statement is indicated in bold (*n* = 100–104, not every question was answered by the gastroenterologists).

Gastroenterologists rated their knowledge about oral diseases with a mean score of 2.20 ± 0.00, on a 5-point Likert scale. Most (58.8%) reported that they had obtained their knowledge about gastrointestinal disease-related oral problems during their specialization, followed by textbooks (43.1%) and scientific articles (28.4%).

### Dentists

A total of 103 dentists returned the questionnaire, resulting in a response rate of 20.6%. Four questionnaires were incomplete and 6 were returned by retired dentists, resulting in 93 questionnaires for statistical analysis. There were 38.7% females and 60.2% males and the average age of the respondents was 48.3 ± 12.8 years (range 25–78 years). The average year of graduation was 1993 ± 13 years ranging from 1967 to 2016. On average, the dentists worked 32.8 ± 7.7 hours per week and the mean number of patients consulted per week was 88.5 ± 44.0. These data are summarized in Table 1.

89.2% of the dentists consulted a general practitioner, an oral and maxillofacial surgeon, or a medical specialist about all kinds of medical issues during the last 5 years; 91.6% a general practitioner, 76.8% an oral and maxillofacial surgeon, 76.8% a cardiologist, and 13% a gastroenterologist. The mean number of contacts with a general practitioner, an oral and maxillofacial surgeon, or a medical specialist during the last 5 years was 20.7 ± 44.4. Fifty percent of the dentists who consulted a gastroenterologist did this for additional information, 41.7% because of symptoms in the mouth that were possibly related to a gastrointestinal disease, 33.3% because of questions about medication, and 25% to discuss the dental treatment plan (multiple answers were possible). The dentists valuated consultation of gastroenterologists as very useful with a mean score of 4.67 ± 0.49 on a 5-point Likert scale. There were no statistically significant differences with regard to gender (male 4.80 ± 0.45 versus female 4.50 ± 0.55, Mann–Whitney *U*-test, *P* = .353). Dentist indicated that there was not enough contact between gastroenterologists and dentists with a mean score of 2.24 ± 1.05 on a 5-point Likert scale whereby 92% of the dentists felt that communication with gastroenterologists should be improved, preferably through postgraduate courses about gastrointestinal diseases (71.7%), whereas 45.7% suggested to align the curricula of the dental and medical schools more closely.

Table 4 presents the answers of the dentists on 9 statements about gastrointestinal diseases. Dentists answered 50.3% ± 18.7% of the questions correct, 36.4% ± 22.3% of the questions were answered with “don’t know.” There were no statistically significant differences in number of correct answers with regard to gender (Mann–Whitney *U*-test, *P* = .546). Table 5 shows the answers from the dentists on 10 statements about possible oral manifestations of gastrointestinal diseases. Dentists answered 26.6% ± 20.5% of the questions correct, 50.5% ± 26.1% of the questions were answered with “don’t know.” There were no statistically significant differences in number of correct answers with regard to gender (Mann–Whitney *U*-test, *P* = .853).

**Table 4.** Answers of dentists (*n* = 93) on statements about gastrointestinal diseases.

	True	False	Don’t know
The first symptoms of Crohn’s disease and ulcerative colitis often start at early and late adulthood.	**67 (72.0%)**	10 (10.8%)	16 (17.2%)
Clinical symptoms of Crohn’s disease and ulcerative colitis include abdominal pain, diarrhea, and rectal blood loss.	**79 (84.9%)**	4 (4.3%)	10 (10.8%)
Smoking protects against developing ulcerative colitis.	**16 (17.2%)**	36 (38.7%)	41 (44.1%)
Patients with long lasting ulcerative colitis have an increased risk of developing colorectal cancer.	**69 (74.2%)**	2 (2.2%)	22 (23.7%)
Celiac disease patients can have diarrhea based on malabsorption.	**67 (72.0%)**	5 (5.4%)	21 (22.6%)
Prednisone is used in the treatment of celiac disease.	**19 (20.4%)**	32 (34.4%)	42 (45.2%)
Gastrointestinal fistulas can occur in patients with Crohn’s disease.	**36 (38.7%)**	17 (18.3%)	40 (43.0%)
Crohn’s disease can lead to failure to thrive in children.	**26 (27.9%)**	12 (12.9%)	55 (59.1%)
Peutz–Jeghers syndrome is an inherited condition.	**31 (33.3%)**	2 (2.2%)	60 (64.5%)

The correct answer to the statement is indicated in bold.

**Table 5.** Answers of dentists (*n* = 93) on statements about possible oral manifestations of gastrointestinal diseases.

	True	False	Don’t know
Prevalence of dental caries is higher in patients with inflammatory bowel disease.	**31 (33.3%)**	26 (27.9%)	36 (38.7%)
Pyostomatitis vegetans is more frequently associated with ulcerative colitis than with Crohn’s disease.	**21 (22.6%)**	5 (5.4%)	67 (72.0%)
“Cobblestone” appearance of the buccal mucosa in the mouth is a clinical symptom of ulcerative colitis.	19 (20.4%)	**12 (12.9%)**	62 (66.7%)
Linear ulcerations can occur in Crohn’s disease patients.	**34 (36.6%)**	7 (7.5%)	52 (55.9%)
The prevalence of gingivitis and periodontitis is higher in ulcerative colitis patients compared to the normal population.	**42 (45.2%)**	9 (9.7%)	42 (45.2%)
Diffuse labial and buccal swelling can occur in Crohn’s disease patients.	**22 (23.7%)**	10 (10.8%)	71 (76.3%)
Halitosis and altered taste change are common in Celiac disease patients.	28 (30.1%)	**12 (12.9%)**	53 (56.9%)
Dental enamel defects are common in Celiac disease patients.	**19 (20.4%)**	23 (24.7%)	51 (54.8%)
Aphthous ulcerations are more common in Celiac disease patients compared to Crohn’s disease patients.	17 (18.3%)	**20 (21.5%)**	56 (60.2%)
Oral characteristics of Peutz–Jeghers syndrome are distinct mucocutaneous pigmentations on the lips and oral mucosa.	**34 (36.6%)**	3 (3.2%)	56 (60.2%)

The correct answer to the statement is indicated in bold.

Dentists rated their knowledge about gastrointestinal diseases with a mean of 2.10 ± 1.07 on a 5-point Likert scale. Most dentists (73.1%) reported that they had obtained their knowledge about gastrointestinal diseases during their studies at dental school, followed by textbooks (26.9%) and scientific articles (20.4%). Dentists who reported that they obtained their knowledge from scientific articles rated their knowledge about gastrointestinal diseases significantly higher than dentists who had used other sources (2.53 ± 0.96 versus 1.99 ± 1.08, Mann–Whitney *U*-test *P* = .016) whereas dentists who obtained their knowledge from textbooks had higher scores for both questions about gastrointestinal diseases in general (56.0% ± 16.2%) and questions about gastrointestinal diseases with possible oral manifestations (39.6% ± 17.4%).

## Discussion

The results of this study show that the knowledge and the frequency, extent and content of the interdisciplinary communication between gastroenterologists and dentists in the Netherlands about gastrointestinal diseases with oral manifestations are limited and that there is a need for additional and adequate education. Limited knowledge of oral diseases is not restricted to gastroenterologists, but has previously also been reported for other medical specialists. Pediatricians and general practitioners are reported to have moderate awareness and knowledge of the signs and symptoms of common oral diseases.^14,15^ A recent study from London showed that foundation year 1 doctors and general practitioner trainees lacked knowledge and confidence regarding the management of oral health issues or signposting patients appropriately, and they acknowledged that there is a need to know more about oral health.^16^ This may be because education on oral health is probably not part of the standard curriculum of medical schools in many countries. Most dentists (73.1%) reported that they had obtained their medical knowledge during dental school. This is in line with a study performed in the Netherlands where 91% of the dentists stated that they had obtained most of their medical knowledge during dental school.^17^ The results presented in this study indicate that more postgraduate courses on gastrointestinal diseases and probably also on other medical topics as well are recommended for dentists.

The interdisciplinary communication between gastroenterologists and dentists was valuated as very useful, but both gastroenterologists and dentist felt that it needs improvement. Aligning of medical and dental schools more closely and postgraduate courses was recommended by many respondents. An alternative could be development of e-learning modules, since a study demonstrated that traditional learning versus e-learning did not differ in improvement of healthcare professionals behaviors, skills, or knowledge.^18^

The statement “Oral characteristics of the Peutz-Jeghers syndrome are distinct mucocutaneous pigmentations on the lips and oral mucosa” was answered correct by 93.3% of the gastroenterologists. This might be related to the fact that the mucocutaneous pigmentations occurs in over 95% of individuals with the Peutz–Jeghers syndrome and are only missing in rare cases.^19,20^ Therefore, the mucocutaneous pigmentations are easily observed by gastroenterologists during every visit of the patient. In addition, this syndrome was first described in 1921 by the Dutch internist Jan Peutz, which might also explain why this disease is well known among gastroenterologists in the Netherlands.^21^

As far as we know, this is the first study that assessed the knowledge of gastroenterologists and dentists about gastrointestinal diseases with oral manifestations and the frequency, extend and content of their interdisciplinary communication. Although the response rate in this study was relatively low (20.5% for gastroenterologists and 20.6% for dentists), the respondents were representative for gastroenterologists and dentists in the Netherlands with regard to age and gender.^22^ The relatively low response rate could have introduced a bias in the results of our study since gastroenterologists and dentists that are interested in oral manifestations of gastrointestinal diseases and who have communicated in the past with each other might have been more motivated to complete the questionnaire. Another limitation is that the results reflect the situation in the Netherlands and may not apply to gastroenterologists and dentists in other countries.

## Conclusion

This study suggests that the knowledge of gastroenterologists and dentists about gastrointestinal diseases with oral manifestations is limited. Both gastroenterologists and dentists acknowledge this observation and feel that the communication between one another should be improved because both valuated interdisciplinary consultations as very useful for the treatment of their patients. Gastroenterologists and dentists suggest to improve the communication by aligning the medical and dental studies more closely and through postgraduate courses.

## Data Availability

Data not publicly available.
